# Co-overexpression of TGF-β and SOX9 via rAAV gene transfer modulates the metabolic and chondrogenic activities of human bone marrow-derived mesenchymal stem cells

**DOI:** 10.1186/s13287-016-0280-9

**Published:** 2016-02-01

**Authors:** Ke Tao, Janina Frisch, Ana Rey-Rico, Jagadeesh K. Venkatesan, Gertrud Schmitt, Henning Madry, Jianhao Lin, Magali Cucchiarini

**Affiliations:** Institute of Arthritis, Peking University People’s Hospital, Beijing, 100044 P.R. China; Peking University Health Science Center, Beijing, 100191 P.R. China; Center of Experimental Orthopedics, Saarland University Medical Center, Kirrbergerstraße Bldg 37, Homburg/Saar, D-66421 Germany; Department of Orthopaedic Surgery, Saarland University Medical Center, Kirrbergerstr. Bldg 37, Homburg/Saar, D-66421 Germany

**Keywords:** Human mesenchymal stem cells, Recombinant adeno-associated virus, Multiple gene transfer, Transforming growth factor beta, SOX9, Chondrogenesis

## Abstract

**Background:**

Articular cartilage has a limited potential for self-healing. Transplantation of genetically modified progenitor cells like bone marrow-derived mesenchymal stem cells (MSCs) is an attractive strategy to improve the intrinsic repair capacities of damaged articular cartilage.

**Methods:**

In this study, we examined the potential benefits of co-overexpressing the pleiotropic transformation growth factor beta (TGF-β) with the cartilage-specific transcription factor SOX9 via gene transfer with recombinant adeno-associated virus (rAAV) vectors upon the biological activities of human MSCs (hMSCs). Freshly isolated hMSCs were transduced over time with separate rAAV vectors carrying either TGF-β or *sox9* in chondrogenically-induced aggregate cultures to evaluate the efficacy and duration of transgene expression and to monitor the effects of rAAV-mediated genetic modification upon the cellular activities (proliferation, matrix synthesis) and chondrogenic differentiation potency compared with control conditions (*lacZ* treatment, sequential transductions).

**Results:**

Significant, prolonged TGF-β/*sox9* co-overexpression was achieved in chondrogenically-induced hMSCs upon co-transduction via rAAV for up to 21 days, leading to enhanced proliferative, biosynthetic, and chondrogenic activities relative to control treatments, especially when co-applying the candidate vectors at the highest vector doses tested. Optimal co-administration of TGF-β with *sox9* also advantageously reduced hypertrophic differentiation of the cells in the conditions applied here.

**Conclusion:**

The present findings demonstrate the possibility of modifying MSCs by combined therapeutic gene transfer as potent future strategies for implantation in clinically relevant animal models of cartilage defects in vivo.

## Background

Articular cartilage defects are critical problems in orthopedic surgery because this avascular tissue has a restricted ability for self healing in the absence of chondrogenic cells that may contribute to repair processes. Currently available options in the clinics including marrow stimulation techniques (microfracture, pridie drilling, abrasion arthroplasty) to promote the penetration of such cells from the subchondral bone marrow [1, 2], however, do not allow one to reproduce an original cartilage surface in its structure and function, with generation of a fibrocartilaginous repair tissue (type I collagen) of poor mechanical quality instead of the native, highly organized hyaline cartilage (proteoglycans, type II collagen) capable of supporting joint loading and motion [1–4]. While administration of bone marrow-derived mesenchymal stem cells (MSCs), an attractive source of cells for regenerative purposes, has been already attempted in patients to activate the regenerative processes in focal cartilage defects [2, 5–10], the outcomes have not been consistently associated with the formation of functional, hyaline-like repair tissue that fully and stably integrates with the surrounding, intact cartilage.

In this regard, genetic modification of MSCs prior to implantation in sites of cartilage damage might be a potent approach to overcome such issues by enhancing the chondroreparative activities of the cells [11, 12]. Various gene sequences have been tested thus far as potential chondroregenerative candidates, including the cartilage oligomeric matrix protein (COMP), bone morphogenetic proteins (BMPs), transforming growth factor beta (TGF-β), insulin-like growth factor I (IGF-I), basic fibroblast growth factor (FGF-2), the SOX family of transcription factors, zinc finger protein 145 (ZNF145), Indian hedgehog (Ihh), and Wnt11 [13–24]. Yet reports from diverse groups showed that multiple therapeutic gene transfer might be more valuable to stimulate the repair activities in these cells relative to independent treatments [13, 17, 21, 25–27], a finding also described by us in human articular chondrocytes [28].

In the present study, and for the first time to our best knowledge, we evaluated the possibility of codelivering TGF-β and SOX9, two of the most potent chondrogenic factors [29–33], to primary hMSCs as a means to stimulate the chondroreparative activities of such cells as a thorough extension of our previous work using independent application of these agents [22, 23]. Gene delivery was performed using the attractive, clinically adapted recombinant adeno-associated virus (rAAV) vectors that transduce MSCs at very high efficiencies (up to 100 %) and over extended periods of time (at least 3 weeks) without altering their differentiation potential [14, 16, 22, 23]. Of further note, transduction via rAAV does not raise viral interference, allowing for concomitant administration of independent vectors in their targets [28]. Our data show that successful, prolonged co-overexpression of TGF-β and SOX9 via independent gene transfer using this vector class synergically enhances the levels of proliferation, biosynthesis, and chondrogenesis in hMSCs compared with control treatments while delaying undesirable hypertrophic differentiation in vitro. These observations support the concept of modifying MSCs by multiple rAAV vectors as a promising approach for implantation procedures in articular cartilage defects in vivo.

## Methods

### Experimental design

**Fig. 1:**
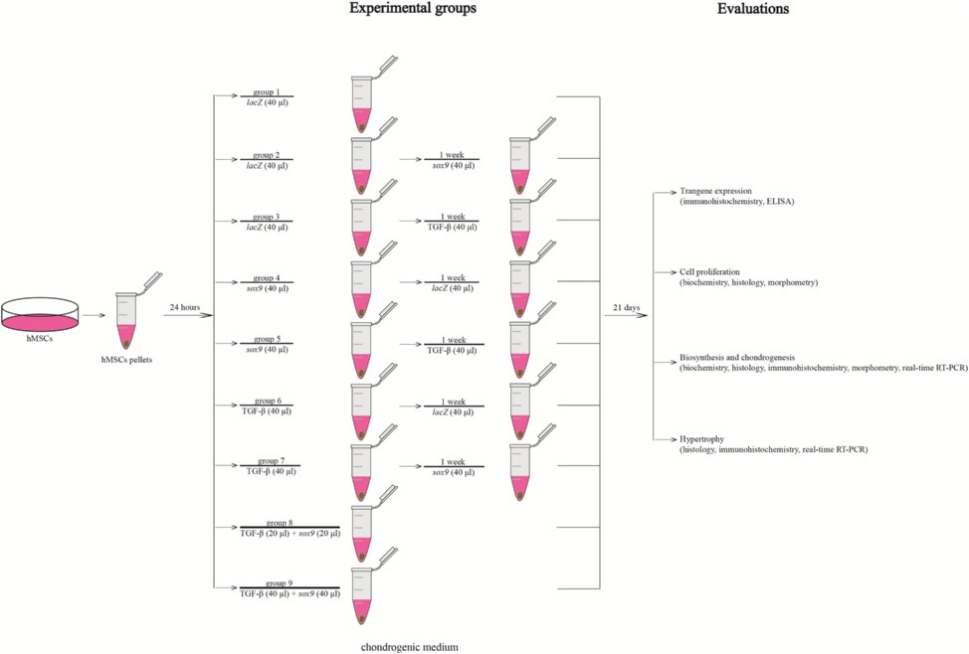
Experimental design. hMSCs were pelleted and divided into nine groups as described in Methods. Group 1, rAAV-*lacZ* (40 μl); group 2, rAAV-*lacZ* (40 μl), rAAV-FLAG-h*sox9* (40 μl) 1 week later; group 3, rAAV-*lacZ* (40 μl), rAAV-hTGF-β (40 μl) 1 week later; group 4, rAAV-FLAG-h*sox9* (40 μl), rAAV-*lacZ* (40 μl) 1 week later; group 5, rAAV-FLAG-h*sox9* (40 μl), rAAV-hTGF-β (40 μl) 1 week later; group 6, rAAV-hTGF-β (40 μl), rAAV-*lacZ* (40 μl) 1 week later; group 7, rAAV-hTGF-β (40 μl), rAAV-FLAG-h*sox9* (40 μl) 1 week later; group 8, rAAV-hTGF-β (20 μl) concomitant with rAAV-FLAG-h*sox9* (40 μl); group 9, rAAV-hTGF-β (40 μl) concomitant with rAAV-FLAG-h*sox9* (40 μl). Cultures were maintained for 21 days in chondrogenic medium for further evaluations. *ELISA* enzyme-linked immunosorbent assay, *hMSC* human bone marrow-derived mesenchymal stem cell, *TGF-β* transforming growth factor beta

Human bone marrow-derived mesenchymal stem cells (hMSCs) were pelleted (2 × 10^5^ cells/pellet) and kept in chondrogenic medium [13, 14, 16, 17, 19–24] for 24 hours prior to transduction. The hMSC pellets were next treated with the various rAAV vectors or vector combinations according to the following nine conditions for maintenance in chondrogenic medium over a period of 21 days (Fig. 1): group 1, pellets transduced with 40 μl rAAV-*lacZ*; group 2, pellets immediately transduced with 40 μl rAAV-*lacZ* and 1 week later with 40 μl rAAV-FLAG-h*sox9*; group 3, pellets immediately transduced with 40 μl rAAV-*lacZ* and 1 week later with 40 μl rAAV-hTGF-β; group 4, pellets immediately transduced with 40 μl rAAV-FLAG-h*sox9* and 1 week later with 40 μl rAAV-*lacZ*; group 5, pellets immediately transduced with 40 μl rAAV-FLAG-h*sox9* and 1 week later with 40 μl rAAV-hTGF-β; group 6, pellets immediately transduced with 40 μl rAAV-hTGF-β and 1 week later with 40 μl rAAV-*lacZ*; group 7, pellets immediately transduced with 40 μl rAAV-hTGF-β and 1 week later with 40 μl rAAV-FLAG-h*sox9*; group 8, pellets cotransduced with 20 μl rAAV-hTGF-β and 20 μl rAAV-FLAG-h*sox9*; and group 9, pellets cotransduced with 40 μl rAAV-hTGF-β and 40 μl rAAV-FLAG-h*sox9*.

### Chemicals and reagents

All reagents were from Sigma (Munich, Germany) unless otherwise indicated. Recombinant FGF-2 and TGF-β3 were purchased at Peprotech (Hamburg, Germany). The dimethylmethylene blue dye was from Serva (Heidelberg, Germany). The anti-TGF-β (V) and anti-SOX9 (C-20) antibodies were from Santa Cruz Biotechnology (Heidelberg, Germany), the anti-type II collagen (II-II6B3) antibody from the NIH Hybridoma Bank (University of Iowa, Ames, IA, USA), the anti-type I collagen (AF-5610) antibody from Acris Antibodies (Hiddenhausen, Germany), and the anti-type X collagen (COL-10) antibody from Sigma. Biotinylated secondary antibodies and the ABC reagent were purchased at Vector Laboratories (Alexis Deutschland GmbH, Grünberg, Germany). The TGF-β enzyme-linked immunosorbent assay (hTGF-β1 Quantikine ELISA) was from R&D Systems (Wiesbaden, Germany).

### Cell culture

Bone marrow aspirates (~15 ml) were obtained from the distal femurs of osteoarthritic female and male patients undergoing total knee arthroplasty (*n* = 6) (age 69–76 years). The study was approved by the Ethics Committee of the Saarland Physicians Council. All patients provided informed consent before inclusion in the study and all procedures were in accordance with the Helsinki Declaration. hMSCs were isolated and expanded in culture according to standard protocols [16, 22, 23]. Briefly, aspirates were washed in Dulbecco’s modified Eagle’s medium (DMEM) and centrifuged, and the pellet was resuspended in Red Blood Cell Lysing Buffer in DMEM (1:1). The resulting fraction was washed, pelleted, and resuspended in DMEM containing 10 % fetal bovine serum with 100 U/ml penicillin and 100 μl/ml streptomycin (growth medium). Cells were plated in T75 flasks and maintained at 37 °C in a humidified atmosphere with 5 % CO_2_. The medium was exchanged after 24 hours and every 2–3 days thereafter using growth medium with recombinant FGF-2 (1 ng/ml). Cells were detached and replated for further experiments at the appropriate densities. hMSCs were analyzed by flow cytometry for expression of stem cell surface markers (CD71^+^, CD105^+^, CD34^–^) [16, 22, 23]. All experiments were performed with cells at no more than passage 2.

### Plasmids and rAAV vectors

The constructs were all derived from the same parental adeno-associated vector 2 genomic clone, pSSV9 [34, 35]. rAAV-*lacZ* carries the *lacZ* gene encoding *Escherichia coli* β-galactosidase under the control of the cytomegalovirus immediate-early (CMV-IE) promoter. rAAV-hTGF-β carries a 1.2 kb human active transforming growth factor beta 1 (hTGF-β1) cDNA fragment (Invivogen, Toulouse, France) and rAAV-FLAG-h*sox9* a 1.7 kb FLAG-tagged human *sox9* (h*sox9*) cDNA, both cloned in rAAV-*lacZ* in place of *lacZ* [16, 22, 23, 28, 36]. rAAV were packaged as conventional (not self-complementary) vectors in the 293 packaging cell line using Adenovirus 5 and pAd8 for helper functions. Purification, dialysis, and titration of the vectors by real-time PCR were performed as described previously [16, 22, 23, 28, 36], averaging 10^10^ transgene copies/ml (1/500 functional recombinant viral particles) [16, 22, 23, 28, 36].

### rAAV-mediated gene transfer

hMSC aggregate cultures (2 × 10^5^ cells) were prepared and kept for up to 21 days in defined chondrogenic medium (high-glucose DMEM 4.5 g/l, penicillin/streptomycin, 6.25 μg/ml insulin, 6.25 μg/ml transferrin, 6.25 μg/ml selenous acid, 5.35 μg/ml linoleic acid, 1.25 μg/ml bovine serum albumin, 1 mM sodium pyruvate, 37.5 μg/ml ascorbate 2-phosphate, 10^−7^ M dexamethasone, 10 ng/ml TGFβ3) for (co)transduction with rAAV (20 or 40 μl each vector, 4 × 10^5^ or 8 × 10^5^ functional recombinant viral particles, respectively, multiplicity of infection = 2 or 4) [16, 22, 23].

### Transgene expression

To evaluate the production of TGF-β, samples were washed twice and placed for 24 hours in serum-free medium. The culture supernatants were collected and centrifuged to remove debris, and TGF-β secretion was monitored by ELISA [23]. Quantitative measurements were performed on a GENios spectrophotometer/fluorometer (Tecan, Crailsheim, Germany). Transgene (TGF-β, SOX9) expression was also assessed by immunohistochemical analyses using specific primary antibodies, biotinylated secondary antibodies, and the ABC method with diaminobenzidine as the chromogen [16, 22, 23, 28, 36]. To control for secondary immunoglobulins, the samples were processed with omission of the primary antibody. Samples were examined under light microscopy (Olympus BX 45; Olympus, Hamburg, Germany).

### Biochemical assays

hMSC aggregates were collected and digested with papain [16, 22, 23, 28, 36]. The DNA and proteoglycan contents were determined with a fluorimetric assay using Hoechst 22358 and by binding to dimethylmethylene blue dye, respectively [16, 22, 23]. Data were normalized to total cellular proteins using a protein assay (Pierce Thermo Scientific, Fisher Scientific, Schwerte, Germany). All measurements were performed on a GENios spectrophotometer/fluorometer (Tecan).

### Histological and immunohistochemical analyses

hMSC aggregates were harvested, fixed in 4 % formalin, dehydrated in graded alcohols, embedded in paraffin, and sectioned (3 μm). Sections were stained with hematoxylin and eosin (H & E) (cellularity), toluidine blue (matrix proteoglycans), and alizarin red (matrix mineralization) as described previously [16, 22, 23]. Expression of type II/type I/type X collagen was detected by immunohistochemistry using specific primary antibodies as already described [16, 22, 23]. To control for secondary immunoglobulins, sections were processed with omission of the primary antibody. Samples were examined under light microscopy (Olympus BX 45).

### Morphometric analyses

The cell densities on H & E-stained sections, the intensities of toluidine blue and alizarin red staining and those of type II and type I collagen immunostaining (pixels per standardized area), and the percentage of cells positive for type X collagen immunostaining were measured using 10 serial histological and immunohistochemical sections for each parameter, test, and replicate condition using the SIS analySIS program (Olympus), Adobe Photoshop (Adobe Systems, Unterschleissheim, Germany), and Scion Image (Scion Corporation, Frederick, MD, USA) [16, 22, 23].

### Real-time RT-PCR analyses

Total RNA from pellets (*n* = 3) was extracted from the cultures using the RNeasy Protect Mini Kit with an on-column RNase-free DNase treatment (Qiagen, Hilden, Germany). RNA was eluted in 30 μl RNase-free water. Reverse transcription was carried out with 8 μl eluate using the 1^st^ Strand cDNA Synthesis kit for RT-PCR (AMV; Roche Applied Science, Mannheim, Germany). An aliquot of the cDNA product (2 μl) was amplified by real-time RT-PCR using the Brilliant SYBR Green QPCR Master Mix (Stratagene, Agilent Technologies, Waldbronn, Germany) on an Mx3000P QPCR operator system (Stratagene) as follows: initial incubation (95 °C, 10 minutes), amplification for 55 cycles (denaturation at 95 °C, 30 seconds; annealing at 55 °C, 1 minute; extension at 72 °C, 30 seconds), denaturation (95 °C, 1 minute), and final incubation (55 °C, 30 seconds). The primers (Invitrogen, Darmstadt, Germany) used were SOX9 (chondrogenic marker) (forward, 5′-ACACACAGCTCACTCGACCTTG-3′; reverse, 5′-GGGAATTCTGGTTGGTCCTCT-3′), aggrecan (ACAN, chondrogenic marker) (forward, 5′-GAGATGGAGGGTGAGGTC-3′; reverse 5′-ACGCTGCCTCGGGCTTC-3′), type II collagen (COL2A1; chondrogenic marker) (forward, 5′-GGACTTTTCTCCCCTCTCT-3′; reverse, 5′-GACCCGAAGGTCTTACAGGA-3′), type I collagen (COL1A1; osteogenic marker) (forward, 5′-ACGTCCTGGTGAAGTTGGTC-3′; reverse, 5′-ACCAGGGAAGCCTCTCTCTC-3′), type X collagen (COL10A1; marker of hypertrophy) (forward, 5′-CCCTCTTGTTAGTGCCAACC-3′; reverse, 5′-AGATTCCAGTCCTTGGGTCA-3′), and glyceraldehyde-3-phosphate dehydrogenase (GAPDH; housekeeping gene and internal control) (forward, 5′-GAAGGTGAAGGTCGGAGTC-3′; reverse, 5′-GAAGATGGTGATGGGATTTC-3′) (all 150 nM final concentration) [16, 22, 23]. Control conditions included reactions using water and nonreverse-transcribed mRNA. Specificity of the products was confirmed by melting curve analysis and agarose gel electrophoresis. The threshold cycle (Ct) value for each gene of interest was measured for each amplified sample using MxPro QPCR software (Stratagene), and values were normalized to GAPDH expression using the 2^–ΔΔCt^ method, as described previously [16, 22, 23].

### Statistical analyses

Each treatment condition was performed in triplicate in three independent experiments for each patient. Data are expressed as the mean ± standard deviation (SD) of separate experiments. The *t* test and the Mann–Whitney rank-sum test were used where appropriate. Any *P* value <0.05 was considered statistically significant.

## Results

### Effective and sustained TGF-β and *sox9* co-overexpression in chondrogenically-induced hMSC aggregate cultures via combined rAAV-mediated gene transfer

hMSCs were first transduced with the various rAAV vectors and vector combinations in chondrogenically-induced aggregate cultures as presented in Fig. 1 to evaluate the ability of this vector class to promote the co-expression of the chondrogenic TGF-β and *sox9* genes over time in cells committed toward the chondrocyte phenotype compared with control conditions.

**Fig. 2:**
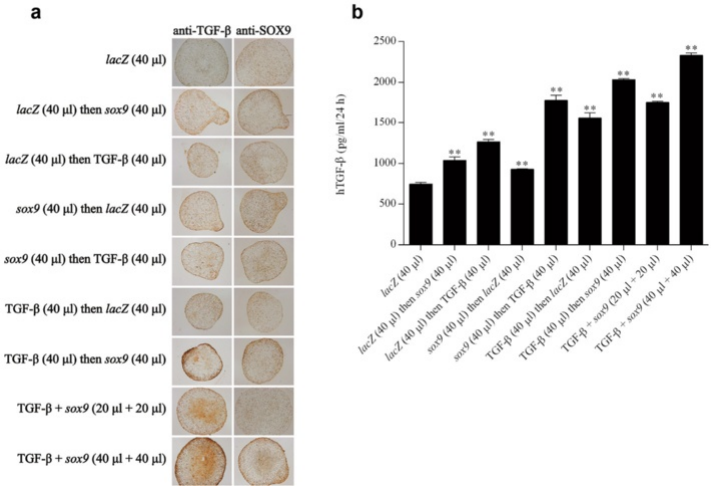
Transgene expression in chondrogenically-induced hMSCs upon administration of rAAV vectors. hMSC aggregates were transduced with the various vectors or vector combinations as described in Fig. 1 and in Methods. Samples were histologically processed after 21 days to detect the expression of TGF-β and SOX9 by immunohistochemistry (magnification × 4; all representative data) **a** and to monitor the production of TGF-β by ELISA **b** as described in Methods. **Statistically significant compared with group 1 (rAAV-*lacZ*) (**P ≤0.010). *TGF-β* transforming growth factor beta

Strong, significant expression of TGF-β was noted for at least 21 days especially when the rAAV-hTGF-β vector was provided to the cultures, as noted by immunohistochemical analysis that revealed the strongest signal upon concomitant TGF-β and *sox9* gene transfer at the highest vector doses applied (40 μl each vector) (Fig. 2a). This observation was corroborated by results of a specific TGF-β ELISA (Fig. 2b), showing an up to 3-fold difference when coapplying the TGF-β and *sox9* vectors at high vector dose (40 μl each vector) compared with the *lacZ* condition (*P* ≤0.010). Strong SOX9 expression was also achieved for at least 21 days in the cultures in the presence of the rAAV-FLAG-h*sox9* vector as noted by immunohistochemistry, also revealing the strongest signal in the simultaneous presence of the TGF-β and *sox9* vectors at high vector dose (40 μl each vector) (Fig. 2b).

### Effects of co-overexpressing TGF-β and *sox9* via rAAV upon the biological activities and differentiation potential of chondrogenically-induced hMSC aggregate cultures

hMSCs were next transduced with the various rAAV vectors and vector combinations in chondrogenically-induced aggregate cultures to examine the potential effects of the concomitant TGF-β and *sox9* expression over time upon the proliferative, metabolic, and differentiation activities in cells compared with control conditions, with a focus on concomitant TGF-β/*sox9* gene transfer at high vector doses based on the findings of optimal transgene co-overexpression.

**Fig. 3:**
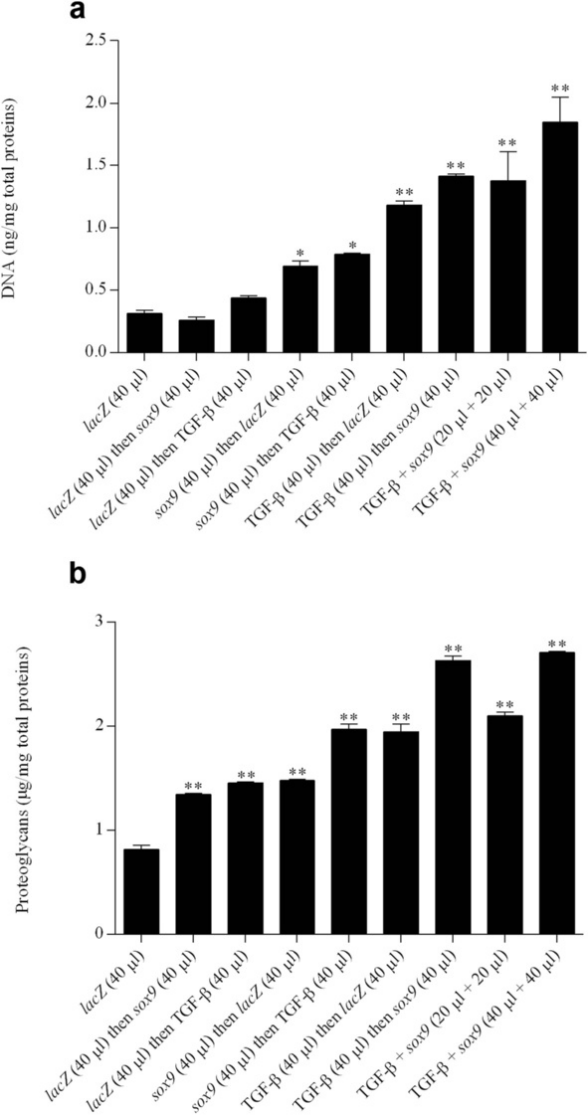
Biochemical analyses in chondrogenically-induced hMSCs upon administration of rAAV vectors. hMSC aggregates were transduced with the various vectors or vector combinations as described in Fig. 1 and in Methods. Samples were processed after 21 days to monitor the DNA **a** and proteoglycan contents **b** as described in Methods. *,**Statistically significant compared with group 1 (rAAV-*lacZ*) (*P ≤0.050, **P ≤0.010). *TGF-β* transforming growth factor beta

**Fig. 4:**
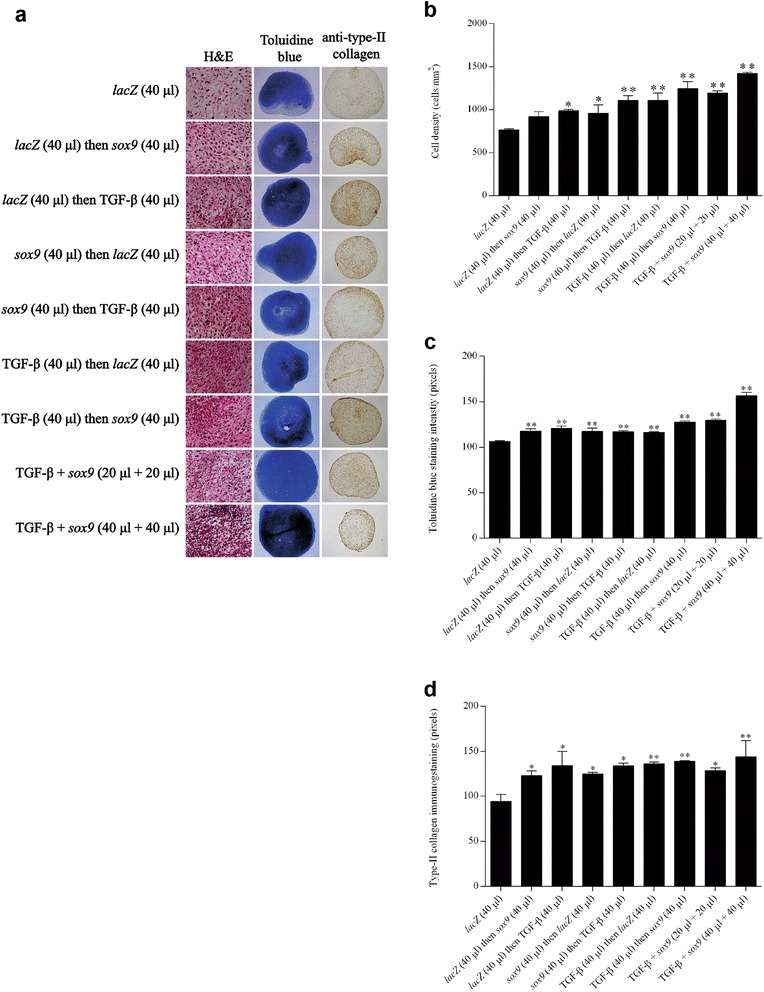
Metabolic and differentiation activities in chondrogenically-induced hMSCs upon administration of rAAV vectors. hMSC aggregates were transduced with the various vectors or vector combinations as described in Fig. 1 and in Methods. Samples were histologically and histomorphometrically processed after 21 days to evaluate cellularity (H & E staining; magnification × 20) **a**, **b** and the deposition of matrix proteoglycans (toluidine blue staining; magnification × 4) **a**, **c** and type II collagen (magnification × 4) **a**, **d** as described in Methods (all representative data). *,**Statistically significant compared with group 1 (rAAV-*lacZ*) (*P ≤0.050; **P ≤0.010). *H & E* hematoxylin and eosin, *TGF-β* transforming growth factor beta

High, significant levels of cell proliferation were noted for at least 21 days especially when rAAV-hTGF-β and rAAV-FLAG-h*sox9* were provided at the highest vector doses tested (40 μl each vector), as noted by an evaluation of the DNA contents in the cultures (7.2-fold difference compared with the *lacZ* condition; *P* ≤0.010) (Fig. 3a) and of the cell densities on H & E-stained histological sections (1.9-fold difference compared with *lacZ*; *P* ≤0.010) (Fig. 4a, b). Elevated, significant levels of matrix synthesis and chondrogenic differentiation were also reported for at least 21 days especially when the TGF-β and *sox9* vectors were administered at the highest vector doses tested (40 μl each vector), as observed by an estimation of the proteoglycan contents in the cultures (3.4-fold difference compared with *lacZ*; *P* ≤0.010) (Fig. 3b) and of the intensities of toluidine blue staining (1.4-fold difference compared with *lacZ*; *P* ≤0.010) (Fig. 4a, c) and of type II collagen immunostaining (1.6-fold difference compared with *lacZ*; *P* ≤0.010) (Fig. 4a, d). These findings were corroborated by the results of a real-time RT-PCR analysis revealing most particularly enhanced levels of chondrogenic SOX9, ACAN, and COL2A1 expression profiles via concomitant TGF-β and *sox9* gene transfer (48-fold, 260-fold, and 23-fold difference compared with *lacZ*; *P* ≤0.010) (Fig. 6).

### Effects of co-overexpressing TGF-β and *sox9* via rAAV upon the hypertrophic differentiation processes in chondrogenically-induced hMSC aggregate cultures

Finally, hMSCs were transduced with the various rAAV vectors and vector combinations in chondrogenically-induced aggregate cultures to determine the possible impact of the concomitant TGF-β and *sox9* expression over time upon hypertrophic events in cells compared with control conditions, again with a focus on the optimal treatment condition.

**Fig. 5:**
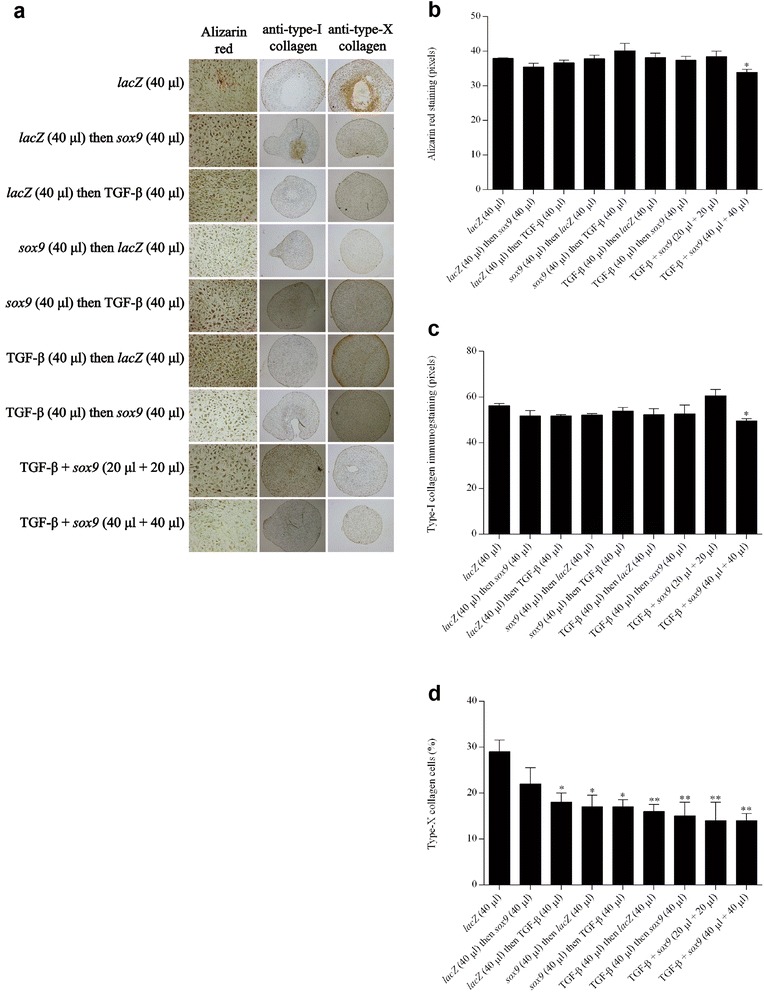
Hypertrophic differentiation in chondrogenically-induced hMSCs upon administration of rAAV vectors. hMSC aggregates were transduced with the various vectors or vector combinations as described in Fig. 1 and in Methods. Samples were histologically and histomorphometrically processed after 21 days to evaluate matrix mineralization (alizarin red staining; magnification × 20) **a**, **b** and the deposition of type I collagen (magnification × 4) **a**, **c** and type X collagen (magnification × 4) **a**, **d** as described in Methods (all representative data). *,**Statistically significant compared with group 1 (rAAV-*lacZ*) (*P ≤0.050; **P ≤0.010). *TGF-β* transforming growth factor beta

**Fig. 6:**
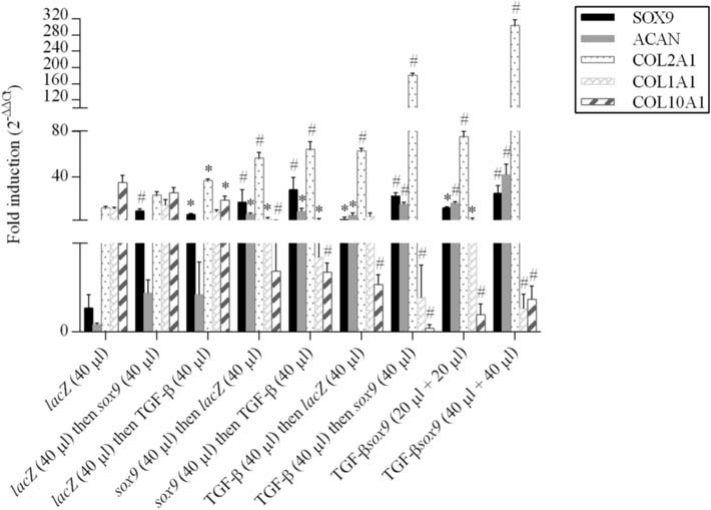
Expression analyses in chondrogenically-induced hMSCs upon administration of rAAV vectors. hMSC aggregates were transduced with the various vectors or vector combinations as described in Fig. 1 and in Methods. Samples were processed after 21 days to monitor the gene expression profiles by real-time RT-PCR as described in Methods. The genes analyzed included the transcription factor SOX9, aggrecan (*ACAN*), type II collagen (*COL2A1*), type I collagen (*COL1A1*), and type X collagen (*COL10A1*), with GAPDH serving as a housekeeping gene and internal control. Threshold cycle (*Ct*) values were obtained for each target and GAPDH as a control for normalization, and fold inductions (relative to *lacZ*-treated pellets) were measured using the 2^–ΔΔCt^ method. *,^#^Statistically significant compared with rAAV-*lacZ* (*P ≤0.050; ^#^ *P* ≤0.010). *TGF-β* transforming growth factor beta

Remarkably, coadministration of rAAV-hTGF-β and rAAV-FLAG-h*sox9* at the highest vector doses tested (40 μl each vector) significantly decreased the levels of hypertrophic differentiation for at least 21 days in the cultures, as noted by an evaluation of the intensities of alizarin red staining (1.2-fold difference compared with *lacZ*; *P* ≤0.050) (Fig. 5a, b), of type I collagen immunostaining (1.2-fold difference compared with *lacZ*; *P* ≤0.050) (Fig. 5a, c), and of type X collagen immunostaining (2.9-fold difference compared with *lacZ*; *P* ≤0.010) (Fig. 5a, d). Once again, these results were corroborated by findings of a real-time RT-PCR analysis revealing most particularly reduced levels of hypertrophic COL1A1 and COL10A1 expression profiles upon TGF-β and *sox9* co-gene transfer (25-fold and 50-fold difference compared with *lacZ*; *P* ≤0.010) (Fig. 6).

## Discussion

Strategies based on the administration of genetically modified bone marrow-derived MSCs have attracted increased interest in recent years as a means to enhance the healing processes in articular cartilage defects [11, 12]. In this study, we tested the possibility of simultaneously targeting hMSCs to overexpress the chondrogenic TGF-β and SOX9 factors [29–33] by multiple gene transfer using the potent rAAV vectors for a possible synergistic, positive impact on the reparative activities of the cells in vitro.

Our results first indicate that concomitant expression of TGF-β and *sox9* was successfully achieved via independent rAAV gene transfer in hMSCs in vitro for at least 21 days, probably due to good penetration and maintenance of the vectors in these targets, as previously reported when applying these candidate genes as individual treatments to the cells [22, 23]. Combined TGF-β/*sox9* gene transfer allowed for the durable production of TGF-β at levels that were higher than those achieved in the control *lacZ* condition or when providing rAAV-hTGF-β with rAAV-*lacZ* instead of rAAV-FLAG-h*sox9* at comparable vector codoses (1.3-fold to 3-fold more elevated concentrations), possibly due to a regulatory, positive effect of exogenous SOX9 factor upon the expression of TGF-β. More, extensive work on the promoter sequences will be needed to identify possible targets to each factor for trans-expression effects.

The data further show that prolonged, effective co-overexpression of TGF-β and *sox9* was capable of enhancing the levels of cell proliferation, matrix biosynthesis, and chondrogenic differentiation in hMSCs over time in vitro (at least 21 days), concordant with the properties of these agents and with our previous work when rAAV-hTGF-β and rAAV-FLAG-h*sox9* were independently provided to the cells [22, 23, 29–33]. TGF-β/*sox9* coapplication was capable of stimulating these activities in hMSCs to levels higher than those reached in the control *lacZ* condition or when combining each therapeutic sequence with *lacZ* at similar vector codoses (1.2-fold to 7.2-fold more potent proliferative, anabolic, and chondrogenic effects), demonstrating that additive effects could be achieved by simultaneous gene transfer of these two potent factors [37]. Equally important, combined TGF-β/*sox9* delivery advantageously delayed premature hypertrophic differentiation in hMSCs relative to *lacZ* treatment, possibly resulting from antihypertrophic effects of exogenous *sox9* expression [22] that might counterbalance the otherwise prohypertrophic activities of TGF-β [23, 30, 31, 33] in the conditions evaluated here. Interestingly, Liao et al. [38] also reported that exogenous overexpression of *sox9* enhanced the chondrogenic differentiation of MSCs comodified by BMP-2, a member of the TGF-β superfamily, using coadenoviral vector delivery, while inhibiting their hypertrophic differentiation in vitro.

In conclusion, and for the first time to our best knowledge, we provide evidence for the benefits of cotransducing hMSCs via separate therapeutic rAAV vectors to significantly improve their chondroreparative activities in vitro. Work is ongoing to first corroborate the current findings in similar animal cell populations in vitro that may allow one to evaluate the feasibility of translating these findings in experimental orthotopic animal models of articular cartilage defects that provide a natural environment for chondrogenesis [14, 19, 39] in order to confirm that *sox9* expression can counteract possible hypertrophic effects of TGF-β in vivo [40, 41].

## Conclusion

The present findings show the potential of combining stem cell-based and multiple gene-based approaches by administration of independent rAAV gene transfer to interactively stimulate chondroreparative activities of progenitor cells as a means to improve the processes controlling cartilage repair upon future implantation in sites of cartilage injuries.

## Abbreviations

ACAN: Aggrecan
BMP: Bone morphogenetic protein
cDNA: Complementary deoxyribonucleic acid
CMV-IE: Cytomegalovirus immediate-early
COL10A1: Type X collagen
COL1A1: Type I collagen
COL2A1: Type II collagen
COMP: Cartilage oligomeric matrix protein
Ct: Threshold cycle
DMEM: Dulbecco’s modified Eagle’s medium
ELISA: Enzyme-linked immunosorbent assay
FGF-2: Basic fibroblast growth factor
GAPDH: Glyceraldehyde-3-phosphate dehydrogenase
H & E: Hematoxylin and eosin
hMSC: Human bone marrow-derived mesenchymal stem cell
IGF-I: Insulin-like growth factor I
Ihh: Indian hedgehog
MSC: Mesenchymal stem cell
rAAV: Recombinant adeno-associated virus
RT-PCR: Reverse-transcriptase PCR
SD: Standard deviation
SOX: Sex-determining region Y-type high mobility group box
TGF-β: Transforming growth factor beta
Wnt11: Wingless/Int 11
ZNF145: Zinc finger protein 145
